# Discourse Context and Co-Speech Gestures Jointly Shape Hierarchical Prediction During the Processing of a Multimodal Narrative

**DOI:** 10.1162/NOL.a.276

**Published:** 2026-07-31

**Authors:** James Trujillo, Benjamin Straube, Yifei He

**Affiliations:** Institute for Logic, Language and Computation, The University of Amsterdam, Amsterdam, The Netherlands; Department of Psychiatry and Psychotherapy, Philipps University Marburg, Marburg, Germany

**Keywords:** discourse, fMRI, gesture, narrative, prediction, Sentence Gestalt Model

## Abstract

Understanding one another in daily communication depends on predicting language from prior discourse context and visual signals, such as co-speech gestures. However, it remains unclear how discourse context and gestures jointly shape neural predictions during naturalistic language processing. Here, participants watched multimodal narratives with spontaneously produced gestures during fMRI scanning. Leveraging transformer-based computational modeling, we disentangled linguistic predictability from contextual informativity at the sentence level, and observed that these deconstructed measures engaged neural regions associated with predictive processing across multiple multimodal representational levels. Furthermore, greater gesture availability was associated with attenuated predictability-related responses and weaker context-related effects, revealing a push–pull synergy between gestures and context. Our findings extend hierarchical predictive processing frameworks, demonstrating that gestures and discourse jointly, rather than additively, constrain neural predictions at multiple representational scales. These results demonstrate the critical and dynamically integrated role of multimodal predictive mechanisms in everyday communication.

## INTRODUCTION

Visual signals, such as co-speech manual gestures, form a vital part of language. When speakers communicate with one another, such as having a conversation or telling a story, we use not only speech but also visual signals to convey meaning. They commonly reinforce spoken content, helping listeners interpret and predict upcoming information in real time. As a listener, our understanding of what is being conveyed depends on the array of multimodal signals unfolding in the moment (e.g., speech, facial expression, manual gestures) as well as our prior knowledge, including our general world knowledge, but also the immediate discourse context. Investigating how communicatively relevant visual signals, such as manual gestures, and discourse context influence the neural processing of narratives would provide insights into the multimodal, contextually embedded nature of language processing.

Manual co-speech gestures, as one form of multimodal communication, play a key part in face-to-face language (Holler & Levinson, 2019; Özyürek, 2014; Skipper et al., 2009; Vigliocco et al., 2014). Manual co-speech gestures (henceforth: gestures) can emphasize, clarify, and add meaning to what is conveyed in the vocal modality, and can also support language comprehension by providing predictive information about upcoming speech (ter Bekke et al., 2024). While gestures contribute to the effective processing of language, the discourse context also plays an important role. In the case of narratives, our understanding of what is being told is continuously shaped by what came before. This discourse context can shape the interpretation of unfolding words and sentences (Hald et al., 2007; Shuang et al., 2015), supporting the prediction of upcoming words (Otten & Van Berkum, 2008) by constraining semantic predictions based on what would fit the narrative so far. This concept of semantic constraints can also be applied to longer stretches of information, as in the Sentence Gestalt Model (SGM) of language processing (Rabovsky et al., 2018). The SGM, which has primarily been studied in a unimodal fashion, frames language comprehension as a process of updating a larger (e.g., sentence- or discourse-level) “representation”. More recently, this theoretical model has been expanded to encapsulate multimodal, socially embedded language (Trujillo & Holler, 2023). In this framework, both words and visual signals (such as co-speech gestures) act as evidence for updating the representation. However, there is currently no empirical evidence for how gestures and discourse work together to inform predictive language processing.

In line with behavioral results, neuroimaging also shows that the presence of meaningful, but not unrelated (Green et al., 2009) gestures contribute to the processing of speech semantics at the neural level (Drijvers et al., 2021; He et al., 2018; Zhang et al., 2021). The integration of speech and gesture can be seen in activation of the left posterior superior temporal sulcus (pSTS) and middle temporal gyrus (MTG), and left inferior frontal gyrus (IFG) (Dick et al., 2014; Drijvers et al., 2021; He et al., 2015; Weisberg et al., 2017). Importantly, both mismatching and unrelated gestures (Green et al., 2009; Willems et al., 2007) as well as contextually less-likely words (Wang et al., 2023) elicit greater activation of the MTG and IFG. This may be due to a greater updating or integration cost for less well-fitting information. A recent computational framework also posits the pSTS as a key area for multimodal integration and the perception of meaningful representations of meaningful multimodal Gestalts (i.e., ensembles of auditory and/or visual signals; Benetti et al., 2023). However, comprehension during daily communication often involves not only integration at a local level, but also active inferencing of upcoming information by jointly leveraging gestures and the broader discourse context.

Understanding how gestural and contextual information interact in more extended, naturalistic settings requires us to systematically quantify these influences. For this, we can turn to information theoretic measures of the informativeness of individual words, such as *surprisal* (Aurnhammer & Frank, 2019; Hale, 2001; Levy, 2008). Surprisal captures the unexpectedness of a word given its immediate context, and serves as a proxy for the effortfulness of processing the word. From a unimodal domain, higher surprisal words are known to elicit a greater n400 effect (Frank et al., 2013) and greater activation in the bilateral anterior temporal lobe and left IFG (Bhattasali & Resnik, 2021; Willems et al., 2016), which may reflect the integration of information. In a multimodal setting, there is evidence that gestures reduce the n400 effect associated with word frequency and surprisal (Osorio et al., 2024; Zhang et al., 2021). These emerging applications of the information-theoretic approach suggest that the multimodal embedding of high-surprisal words reduces the cognitive cost of updating.

The current study combines linguistic predictability, contextual informativeness, and co-speech gestures to assess the neural processing of multimodal discourse. Specifically, we ask how the presence or absence of gestures influences brain activation associated with (a) decreasing linguistic predictability, and (b) decreasing contextual informativeness. Together, these questions directly assess if co-speech gestures—together with accumulating discourse context—jointly shape predictive processes during multimodal narrative comprehension, leading to a multimodal, discourse-enriched extension of the predictive language processing framework that aligns with real-world communication.

## METHODS

### Participants

Twenty right-handed, native speakers of German participated in the study (seven female). Their mean age was 25.4 years, ranging between 22 and 35 years. All participants gave written, informed consent before participating and reported normal or corrected-to-normal vision. None reported either hearing deficits or relevant medical or psychiatric conditions. The study protocol was assessed and approved by the local ethics committee Philipps University Marburg, Germany.

### Stimuli and Procedure

**Figure 1.**
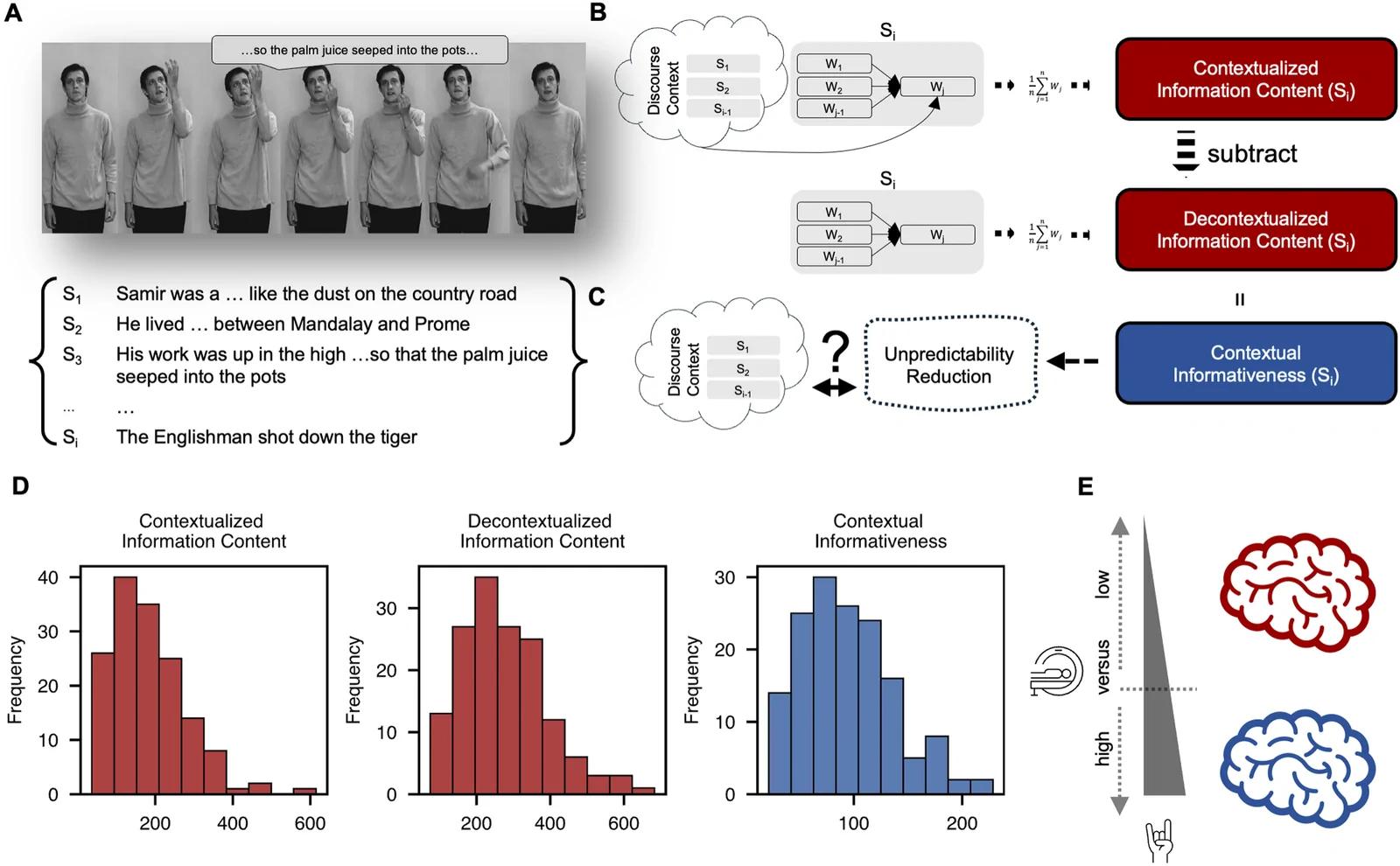
fMRI data analysis pipeline. (A) fMRI data were collected when participants watched a naturalistic multimodal story with the actor of the story narrating sentences in a story auditorily (lower panel) and simultaneously producing a large number of co-speech gestures (upper panel, cf., Cuevas et al., 2019). (B) We computed unpredictability measure (information content) for each sentence (S_i_) by either conditioning based on the entire discourse (contextualized information content, upper panel) or not (decontextualized information content, lower panel). (C) The difference between both measures results in contextual informativeness, which reflects how much predictability is increased by the discourse context. (D) The distributions of these three measures. (E) The three measures were entered into a parametric fMRI analysis for low- versus high-gesture conditions. A direct comparison between low- and high-gesture conditions reveals how gestures and discourse context jointly modulate neural language processing.

Participants watched 16 video clips of a professional actor who narrates an adapted version of the story *Der Kuli Kimgun* as naturally as possible while producing a large number of co-speech gestures (individual clips lasting between 1:02 and 3:31 minutes, story duration = 32:12 minutes; Figure 1A). The story was unfamiliar to all participants. Consent was obtained from the actor to use his image for research and publication purposes. Throughout the story, the actor performs a total of 493 various types of co-speech hand gestures (Cuevas et al., 2019; Osorio et al., 2024). We then calculated, for each sentence, the number of gestures that were co-presented (*M* = 3.84, *SD* = 2.98), and carried out a median split that categorized all sentences as either low-gesture or high-gesture, indicating the amount of accompanying gestures. The number of different types of gestures that occurred in both high and low conditions is reported in Supporting Information Table S1 (Supporting Information can be found at https://doi.org/10.1162/NOL.a.276).

During the acquisition of fMRI data, participants were asked to watch and listen to the videos presented through a mirror mounted on the head coil without engaging in any online tasks. Air conduction headphones (Siemens) were used to present verbal information. The loudness of the headphones was kept constant across sessions. To ensure that participants listened to the narrative, they were asked to answer a questionnaire containing details of the story (e.g., critical moments, narrative shifts, etc.).

### Computational Modeling

We calculate three related measures of linguistic information at the sentence level, following the work of Giulianelli and Fernández (2021): *contextualized information content*, *decontextualized information content*, and *context informativeness* (Figure 1B and C). These calculations build upon the widely applied measures of *surprisal* (Aurnhammer & Frank, 2019; Hale, 2001; Levy, 2008). Word surprisal is defined as the negative log probability of a word conditioned on some context. Often, this was done using an *n-gram* approach (Frank et al., 2013; Willems et al., 2016), wherein the “context” is the preceding two words (i.e., in the case of trigrams), or with a relatively local context. In other words, the frequency of co-occurrences of sequences of words from a large training corpus is used to calculate the probability of a given word occurring. The current approach uses a powerful transformer-based model to calculate word probability while using a large context, allowing us to calculate the probability of words conditioned on not only the preceding few words, but also the entire sentence up until that point, or the entire narrative up until that point (Giulianelli & Fernández, 2021). It is important to note that we adopt the term *information content* in the present study to highlight that our approach is not working with individual word surprisal, but rather with length-corrected, sentence-level surprisal conditioned on the entire preceding context (in the case of *contextualized information content*). While Giulianelli and Fernández (2021) used the GPT-2 large language model, we used Mixtral 8x7B, a so-called small-language model that uses a sparse mixture-of-experts architecture (Artetxe et al., 2022), providing a more efficient (i.e., less computationally heavy) model. Mixtral 8x7B has also been benchmarked outperforming models such as GPT-3.5 on many tasks, despite Mixtral’s smaller size (Jiang et al., 2024). Given the small amount of linguistic information being used in the present study (i.e., one narrative), and Mixtral’s baseline performance on multilingual benchmarks, no fine-tuning was performed.

To prepare the data, the transcript for the spoken narrative was split into individual sentences. *Decontextualized information content* was calculated by taking the average of all negative log word probabilities in a sentence, conditioned only on the preceding words in that same sentence. *Contextualized information content* was also calculated by taking the average of all negative log word probabilities in a sentence, but here the word probabilities are conditioned on the entire preceding discourse (i.e., all words spoken in the narrative up until that point). As a result, extending classic word-based measures such as surprisal, our measures generalize these principles to entire sentences, and allow us to examine *global sentence (un)predictability* rather than focusing solely on individual lexical items. We also computed *context informativeness*, by simply calculating the difference between decontextualized and contextualized information content. Context informativeness then captures the extent to which the preceding discourse (i.e., all sentences from the narrative before a given point) contributes to reducing the average surprisal of a given sentence’s words. In sum, these measures therefore provide a deconstruction of language predictability, capturing sentence surprisal that is informed by context, as well as the extent to which context contributes to a reduction in surprisal. We report a summary of the relationships between these metrics, together with sentence duration, and the number of gestures produced per sentence in Supporting Information Figure S1. Their descriptive statistics and statistical comparison results are reported in Supporting Information Table S2.

### fMRI Data Acquisition

Functional MRI data were acquired using a 3-T Siemens MRT Trio scanner. A T2*-weighted echo-planar imaging sequence was used with the following parameters: repetition time = 2 s, echo time = 30 ms, flip angle = 90°, slice thickness = 4 mm, interslice gap = 0.36 mm, field of view = 230 mm, and a matrix size of 64 × 64. The voxel dimensions were 3.6 × 3.6 × 4.0 mm, with 30 axial slices aligned parallel to the AC-PC plane. The scanning protocol included two runs: the first run consisted of 450 functional images (15 min), while the second run included 520 images (17 min 20 s; Cuevas et al., 2019). Simultaneously with fMRI data, additional EEG data were acquired. These data were intended to be used for another research project and will not be further discussed here. The use of an EEG cap was not expected to affect the quality of the BOLD responses (Iannetti et al., 2005).

### fMRI Preprocessing and Data Analysis

We analyzed the MRI images using Statistical Parametric Mapping (SPM12, v7771) as implemented in MATLAB (Mathworks, Shevorn, MA). We discarded the first two columns to minimize T1 saturation effects. All resulting images were registered to the mean image in a two-pass procedure after registering to the first image in the first run. Images were subsequently corrected for slice-timing differences, co-registered to the anatomical volume normalized into MNI space (voxel size = 2 × 2 × 2 m), and smoothed with an 8 mm isotropic Gaussian filter.

Statistical analysis was carried out using a two-level procedure. In the first level, within both the low- and high-gesture conditions, we modeled single-subject BOLD responses for different sentences based on their onsets and respective durations: sentence onsets were used as event regressors, with sentence duration included to account for temporal variability. Six movement parameters of each participant were also included. The hemodynamic response function (HRF) was modeled by the canonical HRF. We modelled three parametric effects of interest for each sentence. Specifically, we used two transformer models to capture (a) contextualized and (b) decontextualized information content, respectively, and measured (c) contextual informativeness as the difference between contextualized and decontextualized information content. These measures were input as linear parametric modulators of interest in three different sets of fMRI analyses. We then performed a standard full-factorial second-level analysis using the contrast images resulting from the first-level analysis.

For the effect of contextualized/decontextualized information content, we directly looked at the effect of its increase (positive correlation). We started by examining this effect separately for the low- and the high-gesture conditions, and in a second step, looked at the differences in the parametric effects in the low-gesture versus high-gesture conditions. Regarding contextual informativeness, we looked at the brain activations of its decrease (negative correlation). Similarly, we looked at this effect separately in both conditions and then evaluated the difference between the two gesture conditions (low > high).

A Monte Carlo simulation of the brain volume of the current study was employed to determine an adequate voxel contiguity threshold. It is suggested that this correction provides sensitivity to smaller effect sizes and also corrects for multiple comparisons across the whole brain volume (Slotnick, 2017). Assuming an individual voxel type 1 error of *p* < 0.001, a cluster extent of 87 contiguous resampled voxels was indicated as necessary to correct for multiple comparisons at *p* < 0.05. Thus, voxels of clusters with at least 87 voxels and a significance level of *p* < 0.001 are reported for all contrasts. All described activation coordinates are located in MNI space. For the anatomical localization of the clusters, we used the AAL toolbox (Tzourio-Mazoyer et al., 2002).

For each ROI identified in the low > high contrast, we extracted the first eigenvariate per participant, and computed paired *t*-tests, Cohen’s *d*, and Bayes factors (BF_10_) between gesture conditions. These statistics are reported in the figure captions as effect-size summaries for the whole-brain clusters.

## RESULTS

### Information Content

**Figure 2.**
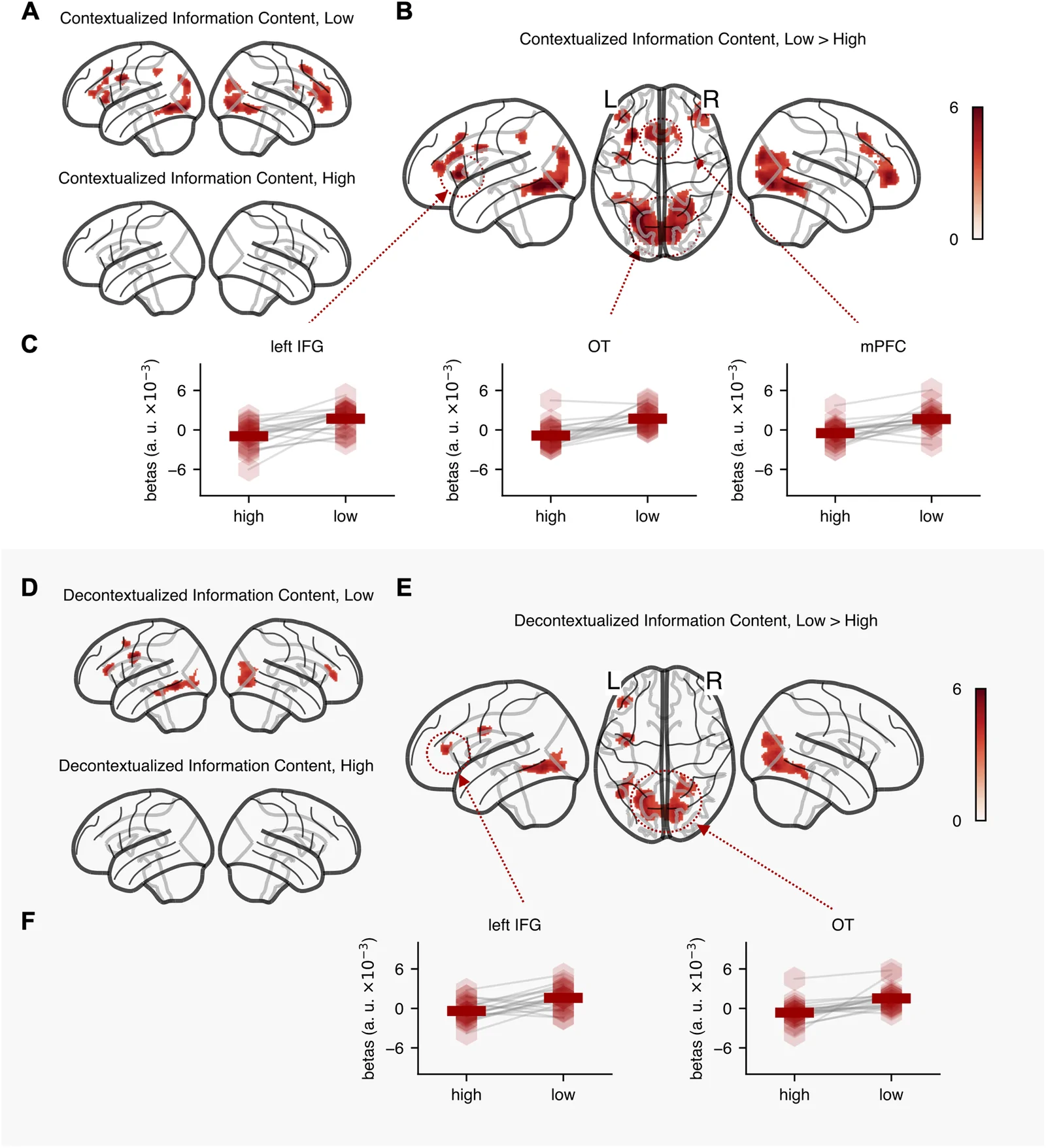
Effects of the contextualized information content (A–C) and decontextualized information content (D–F). (A) Whole brain fMRI results reveal significant clusters in the low-gesture condition (upper) but not in the high-gesture condition (lower) for contextualized information content. (B) Interaction effects (low > high) suggest that gesture presence modulates BOLD activation in a network of clusters, including the bilateral IFG, the OT, and the mPFC. For the glass brain figures, the statistical threshold was set at *p* < 0.001 uncorrected, and only clusters larger than 87 voxels have been included (Monte-Carlo cluster-extent corrected at *p* < 0.05). The colorbar indicates the scale of the t-statistics. (C) Extracted eigenvariates of the respective regions of interest identified in the differential contrast. Importantly, higher beta suggests a **more positive correlation** with contextualized information content. Here, hexagons mark eigenvariates of individual subjects, and the thick bar illustrates the mean across participants. Paired *t*-tests on these eigenvariates confirmed the low > high condition difference in all three ROIs (left IFG: d = 1.37, BF_10_ = 311; OT: d = 1.56, BF_10_ = 653; mPFC: d = 1.27, BF_10_ = 170). (D) Whole brain fMRI results reveal significant clusters in the low-gesture condition (upper) but not in the high condition (lower) for decontextualized information content. (E) Interaction effects (low > high) suggest that gesture presence modulates BOLD activation in clusters including the left IFG and the OT, but not the mPFC. For the glass brain figures, the statistical threshold was set at *p* < 0.001 uncorrected, and only clusters larger than 87 voxels have been included (Monte-Carlo cluster-extent corrected at *p* < 0.05). The colorbar indicates the scale of the t-statistics. (F) Extracted eigenvariates of the respective regions of interest identified in the differential contrast. Importantly, higher beta suggests a **more positive correlation** with decontextualized information content. Here, hexagons mark eigenvariates of individual subjects and the thick bar illustrates the mean across participants. Paired *t*-tests confirmed the condition difference for decontextualized information content in both ROIs (left IFG: d = 1.18, BF_10_ = 36; OT: d = 1.30, BF_10_ = 174). IFG = inferior frontal gyrus; mPFC = medial prefrontal cortex; OT = occipitotemporal gyrus.

Information content of a sentence estimates its unpredictability given the entire preceding discourse (contextualized) or the sentence itself, without preceding discourse (decontextualized). We first looked at the effect of contextualized information content separately in the low- and high-gesture conditions (Figure 2A–C, Supporting Information Table S3). In the low-gesture condition, we observed that increasing contextualized information content led to increased activations within the bilateral IFG and the medial prefrontal cortex (mPFC). Besides, a large cluster is activated in the bilateral occipitotemporal gyrus (OT) that consists of the lateralized occipital temporal gyrus (LOTC), the visual word form area (VWFA), and the fusiform face areas. In the high-gesture condition, no significant clusters positively correlated with contextualized information content. When comparing this parametric effect for low- versus high-gesture conditions (Figure 2B), again, we observed significant effects within the left IFG, the mPFC, and the bilateral OT. To summarize, during the unfolding of a story, when fewer gestures were presented, increased sentence-level unexpectedness (contextualized by prior discourse) led to neural activations in both the lower-order vision and gesture perception areas, as well as in higher order linguistic and social regions including the left IFG and the mPFC. Most importantly, when more gestures were presented, brain activities in these regions were reduced.

Regarding decontextualized information content, in general, the effect is comparable to that of the contextualized information content (Figure 2D–F, Supporting Information Table S4). In the low-gesture condition, we observed that BOLD covaries with this predictor in the left IFG and the OT (Figure 2D; we also observed no significant clusters in the high condition. The difference contrast (Figure 2E) confirms that the left IFG and the OT effects were specific for the low-gesture condition. However, in contrast to contextualized information content, we did not observe a significant effect in the mPFC. In sum, when a sentence’s unexpectedness is not conditioned by prior discourse, its brain correlates are also located in the left IFG and the OT.

### Contextual Informativeness

**Figure 3.**
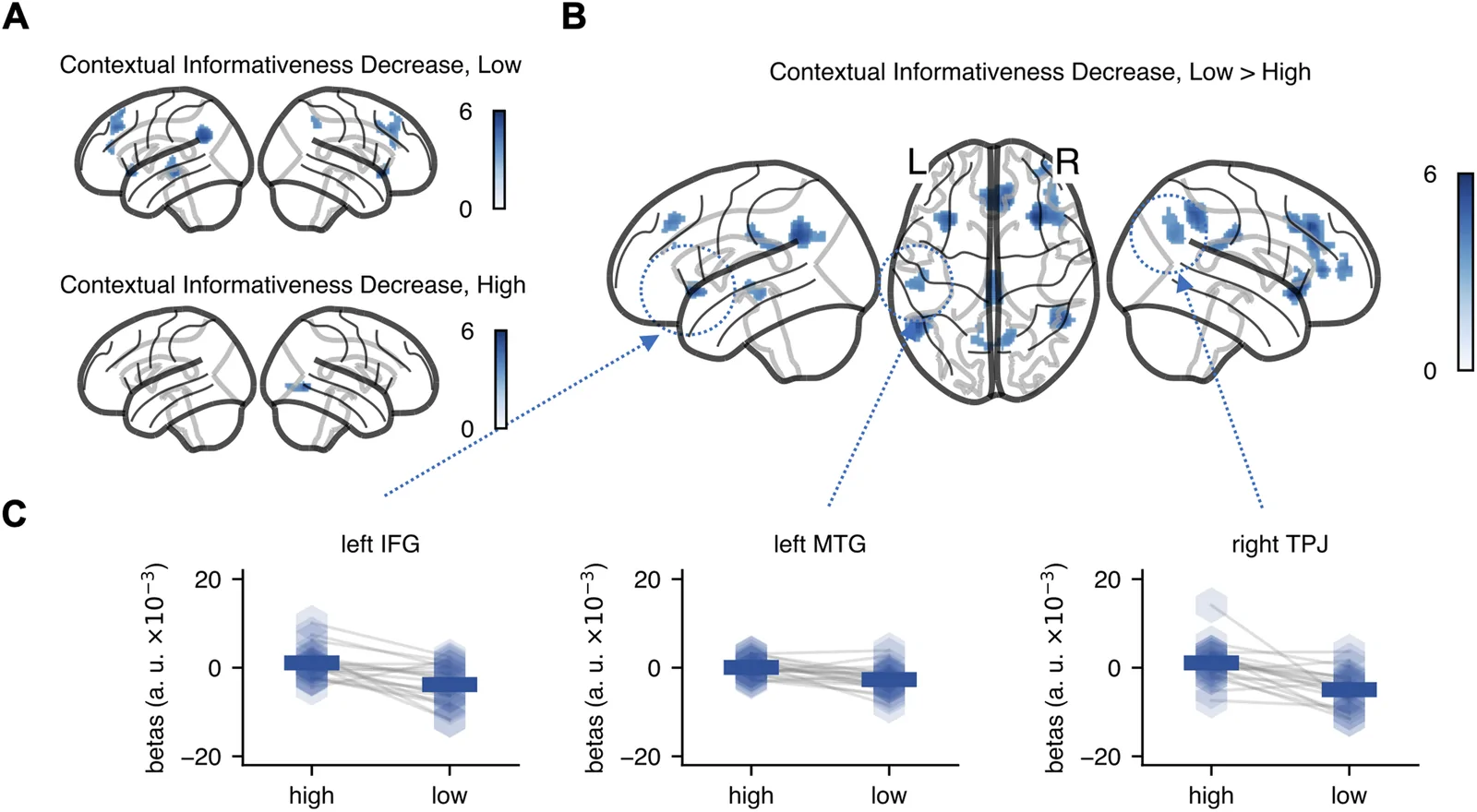
Effects of the decrease in contextual informativeness. (A) Whole-brain fMRI results reveal significant clusters in the low-gesture (upper) and high-gesture (lower) conditions. (B) Differential contrast (low > high) suggests that the effect of contextual informative decrease (negative correlation) was modulated when gestures were present (more positive beta values). This effect was observed in a set of regions including the bilateral IFG, the left MTG, and the bilateral TPJ. For the glass brain figures, the statistical threshold was set at *p* < 0.001 uncorrected, and only clusters larger than 87 voxels have been included (Monte-Carlo cluster-extent corrected at *p* < 0.05). The colorbar indicates the scale of the t-statistics, as resulting from the second-level analysis. (C) Extracted eigenvariates (betas) of the respective regions of interest identified in the interaction analysis. Importantly, a lower beta suggests a **more negative correlation** with contextual informativeness. Here, hexagons mark eigenvariates of individual subjects, and the thick bar illustrates the mean across participants. Paired *t*-tests confirmed the condition difference for contextual informativeness in all three ROIs (left IFG: d = 1.22, BF_10_ = 157; left MTG: d = 1.08, BF_10_ = 24; right TPJ: d = 1.41, BF_10_ = 201). IFG = inferior frontal gyrus; MTG = middle temporal gyrus; TPJ = temporoparietal junction.

Contextual informativeness encodes the extent to which preceding discourse reduces the surprisal of a given sentence. Accordingly, sentences with lower contextual informativeness would require increased brain activity when listening to a story. Thus, in contrast to information content, we therefore tested for negative associations with contextual informativeness (i.e., stronger responses when discourse provides less predictability gain). We first looked at this effect respectively in the low- and high-gesture conditions. In the low condition (Figure 3A, higher, Supporting Information Table S5), we observed significant negative correlations of contextual informativeness in the bilateral IFG, the mPFC, the left MTG, and the bilateral temporoparietal junction (TPJ). In the high condition (Figure 3A, lower, Supporting Information Table S3), we observed a cluster in the right posterior MTG that negatively correlates with contextual informativeness. Interaction analysis revealed significant effects in the bilateral IFG, the bilateral TPJ, the left MTG, and the mPFC (Figure 3B, Supporting Information Table S5). To summarize, for the reduction of contextual informativeness, we primarily observed effects only when fewer gestures were presented (low-gesture condition). These areas consist of regions that are commonly reported to support the processing of discourse and social context (Amodio & Frith, 2006; Kuperberg et al., 2006).

### Matched-Subset Control Analyses

To ensure these effects are not driven by between-condition differences in sentence-level predictors (Supporting Information Table S2), we ran an additional set of parametric analysis, based on high- and low-gesture sentence pairs matched on the target predictor (Welch *p* > 0.07 after matching; Supporting Information Figure S2). The Low > High contrast replicated the pattern observed for contextualized information content (bilateral IFG, OT, mPFC; Supporting Information Figure S3A, Supporting Information Table S6) and for contextual informativeness (bilateral IFG, left MTG, right TPJ; Supporting Information Figure S4, Supporting Information Table S8), but was attenuated for decontextualized information content (Supporting Information Figure S3B, Supporting Information Table S7), indicating that the DIC condition effect is partly carried by raw sentence-level properties and should be interpreted more cautiously than the IC and CI effects.

## DISCUSSION

The current study sought to disentangle the contributions of co-speech gestures and discourse context during predictive language processing. This was done using information content, a deconstructed measure of contextualized sentence-level surprisal to inform analysis of fMRI data from a naturalistic narrative listening experiment. We found that left IFG and OT (amongst others), are sensitive to surprisal when gestures are less present. Other areas, including mPFC, are most sensitive to contextually informed surprisal. Finally, areas such as left IFG, left MTG, and right TPJ were inversely associated with the informativity of prior context, and this relationship was further modulated by the presence of gestures.

### Linguistic Predictability in a Multimodal Context

Both contextualized and decontextualized information content were positively associated with the bilateral IFG and OT. In this case, the increased activation of the IFG may be similar to the reported correlation between IFG activity and semantic processing demands during narrative comprehension (Rodd et al., 2005; Thye et al., 2023). Crucially, we found a stronger correlation between IFG activity and information content when fewer gestures were present. This finding echoes the reported neural facilitation of gestures on surprisal-induced N400 on the single-word level (Osorio et al., 2024; Zhang et al., 2021), suggesting that sentence-level prediction may similarly benefit from the presence of co-speech gestures. More broadly, these findings extend earlier evidence that co-speech gestures engage and possibly shape the brain networks supporting language. Within the multimodal language processing literature employing single sentence paradigms, the left IFG has been reported as a core region of effortful higher level semantic integration (Dick et al., 2014, Drijvers et al., 2021). Here, we observed stronger predictive modulation within the left IFG when gestures are absent, thus further supporting this literature by showing that such gesture-driven modulation operates at the level of sentence-scale discourse prediction.

OT activation has been associated with word surprisal (Frank & Willems, 2017; Takahashi et al., 2021) as well as the processing of actions at different levels of abstraction (Simonelli et al., 2024; Zhuang et al., 2023), with decreased activation when action goals are more predictable (Wurm et al., 2014). Our finding that the OT is sensitive to information content, particularly when there are fewer gestures present, indicates that this region is not just sensitive to sequences unfolding in one particular modality, but rather that this predictive processing is multimodal in nature. The finding of OT being sensitive to linguistically informative *visual* input also suggests that, in addition to the multimodally informed semantic and pragmatic predictions, there are also visual predictions, similar to the phonological or word-level predictions seen in unimodal speech perception.

Together, these findings show different levels of predictions, from lower level (potentially sequential) information in the OT, to higher level social-pragmatic prediction in the mPFC, jointly contributing to render incoming multimodal input more predictable. Co-speech gestures alleviate some of this predictive effort, jointly shaping prediction with discourse context (see below).

### Contextualizing Predictability

A key advance of this study is the use of multimodal data and the application of a deconstructed information measure. In terms of linguistic predictability, previous studies of narrative processing in the brain have often looked at word-level predictability, or static phrasal-level measures (Cuevas et al., 2019). Word-level predictability, while informative, can be challenging to interpret given that different streams of multimodal information unfold at different time-scales (Holler & Levinson, 2019; Trujillo & Holler, 2023), and predictability itself is not evenly distributed within a given utterance (Klafka & Yurovsky, 2021; Trujillo & Holler, 2024, 2025). The approach used here is less vulnerable to word-to-word variation, and is more suitable to examine higher level predictive processes, especially within a multimodal context. Transformer-based (de)contextualized information content (Giulianelli & Fernández, 2021) also provides a powerful measure for language researchers by incorporating arbitrarily long sequences of words as context for this calculation. We can therefore calculate how predictable a word or sentence is in its actual context, but also how informative the context was.

While there is much overlap between the areas associated with contextualized and decontextualized information, the mPFC was only activated for contextualized information content. The mPFC has previously been linked to social prediction (Li et al., 2023) and is sensitive to texts that include a larger narrative context, as opposed to texts with only a shorter sentence context (Xu et al., 2005). The correlation between the mPFC and contextualized information could reflect an updating of longer time-scale representations or predictions of the narrative. This is in line with the framework of multi-scale Gestalt processing (Benetti et al., 2023; Rabovsky et al., 2018; Trujillo & Holler, 2023), and further places the mPFC as a link between these primarily language-oriented frameworks and more general social prediction and belief updating.

While the TPJ has been shown to be sensitive to narrative processing (Haines et al., 2023; Xu et al., 2005), our finding of TPJ activity correlating with contextual informativity suggests a more specific role for the TPJ in narrative comprehension: the integration or prediction of longer time-scale information with currently unfolding information. TPJ activation could therefore relate to the processing of high-level language Gestalts that are framed by discourse and social context and updated by unfolding multimodal information (Trujillo & Holler, 2023). This interpretation also fits well with the studies showing that the TPJ is sensitive to action intentions (Patri et al., 2020; Rizzolatti & Sinigaglia, 2010), which can be seen as larger Gestalts composed of chains of individual actions or movements (Meier et al., 2013). The TPJ may therefore be involved in integrating currently unfolding information with earlier information.

### Unifying Context and Gestures

Our results point to a dynamic interplay between discourse context and gesture in supporting predictive language processing. Specifically, we see that processing the inherent predictability of language is often supported by preceding context that can provide framing or disambiguation. This can be observed in the present results through the reduction in activity in key language areas (e.g., left IFG, left MTG, right TPJ) when context is more informative about the currently unfolding sentence. Importantly, there seems to be a push–pull synergy where both context and co-speech gestures jointly contribute to predictability about unfolding content. However, when there are fewer gestures present, there is a greater gain for discourse context, and a lower gain from context when more gestures are present. Most importantly, from a hierarchical predictive perspective, this synergy directly shapes prediction at various levels of representation from lower-order visual, linguistic to higher order social-pragmatic levels.

### Limitations

Our current study has several limitations. A first limitation is that speech prosody was not quantified, and there was no audio-only condition, which makes it difficult to disentangle the impact of gestures from potential co-occurring modulations in speech prosody. A second limitation is that our three linguistic predictors are correlated by construction, and they also covary with our high- versus low-gesture manipulations. In our analysis, we fit each predictor in a separate GLM rather than jointly: This protects each parametric contrast from the serial-orthogonalization artefacts that arise when correlated predictors share a single SPM model (Mumford et al., 2015), but at the cost of preventing direct partitioning of shared versus unique variance across predictors. We also applied matched-subset analyses to address the interpretational risk that the reported effects might be driven by covariation between a predictor and the gesture manipulation. With our control analysis, we equated predictor distributions across conditions at the stimulus level. However, they do not quantify how much signal each predictor explains independently of the others. A voxel-wise encoding model framework with banded ridge regression and cross-validation (Dupré la Tour et al., 2025) would complement the present approach by providing such a decomposition, though it would require a higher dimensional feature space than the present sentence-level design offers, and thus requires potentially data collection of longer durations.

### Conclusions

Based on naturalistic and multimodal stimulation, our modeling approach on a sentence level provides dynamic measures regarding (a) how prediction operates during the processing of multimodal language, and (b) how this prediction benefits from discourse context and gestures. Our fMRI data provide empirical evidence showing how multimodal prediction operates during comprehension, and how this prediction is supported by both discourse and gestures in an interactive manner. These results broaden the scope of predictive language processing frameworks by showing novel evidence for multimodal prediction in the occipitotemporal gyrus, and a push–pull relationship between contextual information and co-speech gestures in reducing (un) predictability-related brain activation. This study, therefore, provides a deconstructed view of how context and multimodality jointly contribute to the neural processing of naturalistic language.

## FUNDING INFORMATION

Yifei He, Deutsche Forschungsgemeinschaft (https://dx.doi.org/10.13039/501100001659), Award ID: HE8029/2-1, HE8029/5-1. Benjamin Straube, Deutsche Forschungsgemeinschaft (https://dx.doi.org/10.13039/501100001659), Award ID: 533717223. Benjamin Straube, Von-Behring-Röntgen-Stiftung (https://dx.doi.org/10.13039/100009103), Award ID: 59-0002.

## AUTHOR CONTRIBUTIONS

**James Trujillo**: Formal analysis; Writing – original draft; Writing – review & editing. **Benjamin Straube**: Conceptualization; Data curation; Writing – review & editing. **Yifei He**: Conceptualization; Data curation; Formal analysis; Writing – original draft; Writing – review & editing.

## DATA AVAILABILITY STATEMENT

Code to calculate information content and contextual informativity from the transcript data, as well as second-level fMRI contrast images that can be used to explore the results, are available on the Open Science Framework: https://osf.io/yxsrk/?view_only=85c46a24248b40ad90abe5bad1e3f963.

## Supplementary Material


