# Supplementary material for: Discourse Context and Co-Speech Gestures Jointly Shape Hierarchical Prediction During the Processing of a Multimodal Narrative
Source: Neurobiol Lang (Camb). 2026 Jul 31;7:NOL.a.276. doi: 10.1162/NOL.a.276 (PMC13597107; doi:10.1162/NOL.a.276)
Supplement: Supplementary file 1 [file nol-07-276-s001.docx]

**Supplement**

**Figure S1.** Correlation matrix of sentence-related measures. We conducted Pearson’s correlation coefficient between sentence measures, and reported respective statistical significance with asterisks: ***: p < .001, **: p < .01,

*: p < .05, n.s.: not significant.

**Table S1:** Summary of gesture counts for different types of gestures in the high and low gesture conditions

| **Gesture_Type** | **High_Count** | **Low_Count** | **Total_Count** |
| --- | --- | --- | --- |
| **Beat** | 93 | 55 | 148 |
| **Iconic** | 121 | 60 | 181 |
| **Metaphoric** | 54 | 31 | 85 |
| **Deictic** | 48 | 12 | 60 |
| **Emblematic** | 15 | 4 | 19 |
| **Total** | 331 | 162 | 493 |

| **Table S2**: Comparison of sentence measures between high vs. Low gesture conditions. Difference between conditions was statistically evaluated via independent samples t-tests. | | | | |
| --- | --- | --- | --- | --- |
|  |  | High Gesture | Low Gesture |  |
| Contextualized Information Content | Mean | 246.65 | 137.02 | t = 7.95 |
|  | SD | 97.34 | 61.83 | p < .001 |
| Deontextualized Information Content | Mean | 353.94 | 220.17 | t = 8.28 |
|  | SD | 112.34 | 75.98 | p < .001 |
| Contextual Informativeness | Mean | 107.29 | 83.15 | t = 3.54 |
|  | SD | 83.15 | 32.48 | p < .001 |
| Numbers of Gestures | Mean | 6.35 | 1.95 | t = 11.78 |
|  | SD | 2.89 | 1.00 | p < .001 |
| Sentence Duration | Mean | 14.23 | 7.33 | t = 7.98 |
|  | SD | 6.28 | 3.50 | p < .001 |

| **Table S3**: Peak MNI coordinates of clusters showing significant effects of contextualized information content (positive correlation). Cluster-level uncorrected p-value is provided for each cluster. | | | | | | | | |
| --- | --- | --- | --- | --- | --- | --- | --- | --- |
|  |  |  |  | Peak MNI coordinates | | |  |  |
|  | Anatomical Region (Peak) | Hem. | k | x | y | z | t | p (unc.) |
| **Low** |  |  |  |  |  |  |  |  |
|  | Lingual (occipitotemporal) Gyrus | L | 3444 | -18 | -82 | -6 | 6.79 | <.001 |
|  | Precentral Gyrus | L | 372 | -38 | 0 | 30 | 5.82 | .001 |
|  | Insula | L | 354 | -28 | 24 | 4 | 5.09 | .002 |
|  | Middle Frontal Gyrus | R | 797 | 44 | 46 | 4 | 4.99 | <.001 |
|  | SMA | L | 514 | -2 | 20 | 44 | 4.83 | <.001 |
|  | Inferior Frontal Gyrus | R | 108 | 42 | 6 | 24 | 4.00 | .053 |
|  | Inferior Parietal Gyrus | L | 132 | -24 | -44 | 44 | 3.96 | .035 |
| **High** |  |  |  |  |  |  |  |  |
|  | **N. S.** |  |  |  |  |  |  |  |
| **Interaction: Low > High** | |  |  |  |  |  |  |  |
|  | Lingual (occipitotemporal) Gyrus | L | 5275 | -26 | -62 | -6 | 7.67 | <.001 |
|  | Insula | L | 272 | -28 | 24 | 4 | 6.17 | .004 |
|  | Middle Frontal Gyrus | R | 558 | 44 | 48 | 0 | 5.61 | <.001 |
|  | Anterior Cingulate Cortex | L | 659 | -12 | 22 | 28 | 5.12 | <.001 |
|  | Inferior Frontal Gyrus | L | 225 | -36 | 42 | 14 | 4.81 | .008 |
|  | Precentral Gyrus | L | 177 | -38 | 0 | 30 | 4.80 | .017 |
|  | Inferior Parietal Gyrus | L | 155 | -42 | -42 | 44 | 4.25 | .024 |

| **Table S4**: Peak MNI coordinates of clusters showing significant effect of decontextualized information content (positive correlation). Cluster-level uncorrected p value is provided for each cluster. | | | | | | | | |
| --- | --- | --- | --- | --- | --- | --- | --- | --- |
|  |  |  |  | Peak MNI coordinates | | |  |  |
|  | Anatomical Region (Peak) | Hem. | k | x | y | z | t | p (unc.) |
| **Low** |  |  |  |  |  |  |  |  |
|  | Lingual (occipitotemporal) Gyrus | L | 2266 | -24 | -62 | -6 | 5.28 | <.001 |
|  | Precentral Gyrus | L | 195 | -40 | 0 | 30 | 4.90 | .016 |
|  | Inferior Temporal Gyrus | L | 206 | -44 | -56 | -8 | 4.80 | .014 |
|  | Inferior Frontal Gyrus | L | 142 | -40 | 38 | 12 | 4.65 | .035 |
| **High** |  |  |  |  |  |  |  |  |
|  | **N. S.** |  |  |  |  |  |  |  |
| **Interaction: Low > High** | |  |  |  |  |  |  |  |
|  | Precentral Gyrus | L | 421 | -30 | 0 | 30 | 6.02 | <.001 |
|  | Inferior Temporal Gyrus | L | 386 | -44 | -56 | -8 | 5.41 | .002 |
|  | Inferior Frontal Gyrus | L | 201 | -40 | 38 | 12 | 4.78 | .015 |
|  | Lingual (occipitotemporal)  Gyrus | R | 1198 | 14 | -76 | -4 | 4.59 | <.001 |
|  | Superior Frontal Gyrus | L | 98 | -18 | 10 | 48 | 4.47 | .074 |
|  | Middle Frontal Gyrus | R | 177 | 44 | 40 | 8 | 4.12 | .021 |

| **Table S5**: Peak MNI coordinates of clusters showing significant effect of contextual informativeness decrease (negative correlation). Cluster-level uncorrected p-value is provided for each cluster, asterisk indicates significance (p < 0.05) with cluster-level FWE correction. | | | | | | | | |
| --- | --- | --- | --- | --- | --- | --- | --- | --- |
|  |  |  |  | Peak MNI coordinates | | |  |  |
|  | Anatomical Region (Peak) | Hem. | k | x | y | z | t | p (unc.) |
| **Low** |  |  |  |  |  |  |  |  |
|  | Angular Gyrus | L | 432 | -52 | -56 | 30 | 5.48 | .002 |
|  | Insula | R | 186 | 30 | 22 | -10 | 5.02 | .030 |
|  | Middle Temporal Gyrus | L | 409 | -50 | -26 | -8 | 4.98 | .003 |
|  | Superior Frontal Gyrus | L | 532 | -6 | 30 | 64 | 4.83 | .001 |
|  | Insula | L | 162 | -32 | 20 | -6 | 4.66 | .041 |
|  | Middle Frontal Gyrus | R | 203 | 44 | 22 | 42 | 4.35 | .024 |
|  | Anterior Cingulate Cortex | L | 94 | -8 | 42 | 16 | 3.94 | .108 |
|  | Inferior Parietal Gyrus | R | 127 | 46 | -50 | 48 | 3.87 | .066 |
| **High** |  |  |  |  |  |  |  |  |
|  | Middle Temporal Gyrus | R | 259 | 48 | -64 | -2 | 4.87 | .013 |
| **Interaction: Low > High** | |  |  |  |  |  |  |  |
|  | Superior Frontal Gyrus | R | 713 | 8 | 32 | 38 | 5.81 | <.001 |
|  | Insula | R | 430 | 30 | 22 | -8 | 5.75 | .002 |
|  | Angular Gyrus | L | 414 | -52 | -52 | 32 | 5.49 | .003 |
|  | Inferior Parietal Gyrus | R | 406 | 46 | -48 | 46 | 5.21 | .003 |
|  | Insula | L | 163 | -32 | 20 | -6 | 5.05 | .040 |
|  | Middle Frontal Gyrus | R | 301 | 40 | 22 | 38 | 4.54 | .008 |
|  | Middle Cingulate Cortex | R | 305 | 4 | -22 | 32 | 4.47 | .008 |
|  | Superior Frontal Gyrus | R | 113 | 38 | 58 | 12 | 4.32 | .081 |
|  | Middle Temporal Gyrus | L | 120 | -50 | -26 | -8 | 4.12 | .073 |
|  | Precuneus | R | 280 | 14 | -60 | 36 | 4.09 | .010 |

**Figure S2.** Sentence-level predictor distributions for raw and matched-subset sentences. Top: Sentence-level distributions of contextualized information content (IC), decontextualized information content (DIC), and contextual informativeness (CI) in the original high- and low-gesture conditions (n = 65 vs. 87). All three predictors differ significantly between conditions (IC: Welch t = 7.95, p = 2.6 × 10⁻¹²; DIC: t = 8.28, p = 3.9 × 10⁻¹³; CI: t = 3.54, p = 5.9 × 10⁻⁴). Bottom: Distributions after matched-subset selection (IC and DIC: n = 46 per side; CI: n = 63 per side). For each predictor, sentence pairs were selected to control for the target predictor difference across conditions. None of the three predictors differ significantly between conditions after matching (IC: t = 1.81, p = 0.074; DIC: t = 1.81, p = 0.073; CI: t = 1.79, p = 0.076). These matched subsets underlie the supplementary control-analysis fMRI figures (Figures S3 and S4).

**Figure S3.** Contextualized (IC) and decontextualized information-content (DIC) effects after sentence-level matching on each target predictor. (A) Contextualized information content. Here, the Low > High contrast reproduces the bilateral frontotemporal and medial-prefrontal pattern reported in the original analysis, indicating that the effect does not depend on the original between-condition difference. (B) Decontextualized information content. Here, the Low > High contrast is attenuated and shows only small clusters once the between-condition DIC difference is removed. This suggests that the original decontextualized-IC effect is, to some extent, carried by the condition-level mean shift in sentence informativeness. For all analysis, the statistical threshold was set at p < .001 uncorrected, and only clusters larger than 87 voxels have been included (Monte-Carlo cluster-extent corrected at p < .05).

| **Table S6**: Peak MNI coordinates of clusters showing significant effects of contextualized information content in the sentence-level matched subsample (positive correlation). Cluster-level uncorrected p-value is provided for each cluster. | | | | | | | | |
| --- | --- | --- | --- | --- | --- | --- | --- | --- |
|  |  |  |  | Peak MNI coordinates | | |  |  |
|  | Anatomical Region (Peak) | Hem. | k | x | y | z | t | p (unc.) |
| **Low** |  |  |  |  |  |  |  |  |
|  | Lingual (occipitotemporal) Gyrus | L | 4854 | -14 | -80 | -6 | 6.01 | <.001 |
|  | Inferior Parietal Gyrus | R | 1268 | 50 | -38 | 46 | 5.71 | <.001 |
|  | Insula | R | 2048 | 36 | 20 | 2 | 5.54 | <.001 |
|  | Inferior Frontal Gyrus | L | 913 | -34 | 22 | 12 | 5.46 | <.001 |
|  | Anterior Cingulate Cortex | L | 2096 | 2 | 36 | 8 | 5.16 | <.001 |
|  | Precentral Gyrus | L | 404 | -34 | 4 | 34 | 5.02 | .003 |
|  | Inferior Parietal Gyrus | L | 359 | -50 | -46 | 46 | 4.18 | .004 |
|  | Middle Cingulate Cortex | R | 244 | 4 | -28 | 38 | 3.88 | .015 |
| **High** |  |  |  |  |  |  |  |  |
|  | **N. S.** |  |  |  |  |  |  |  |
| **Interaction: Low > High** | |  |  |  |  |  |  |  |
|  | Lingual (occipitotemporal) Gyrus | L | 7578 | -14 | -84 | -6 | 6.80 | <.001 |
|  | Middle Frontal Gyrus | R | 637 | 34 | 44 | -8 | 5.37 | <.001 |
|  | Inferior Frontal Gyrus | L | 165 | -32 | 20 | 10 | 4.87 | .039 |
|  | Anterior Cingulate Cortex | L | 1499 | 0 | 36 | 8 | 4.83 | <.001 |
|  | Inferior Parietal Gyrus | L | 288 | -40 | -54 | 60 | 4.67 | .009 |
|  | Supramarginal Gyrus | R | 807 | 48 | -30 | 30 | 4.65 | <.001 |
|  | Middle Frontal Gyrus | L | 102 | -36 | 56 | 10 | 3.78 | .095 |

| **Table S7**: Peak MNI coordinates of clusters showing significant effects of decontextualized information content in the sentence-level matched subsample (positive correlation). Cluster-level uncorrected p-value is provided for each cluster. | | | | | | | | |
| --- | --- | --- | --- | --- | --- | --- | --- | --- |
|  |  |  |  | Peak MNI coordinates | | |  |  |
|  | Anatomical Region (Peak) | Hem. | k | x | y | z | t | p (unc.) |
| **Low** |  |  |  |  |  |  |  |  |
|  | Lingual (occipitotemporal) Gyrus | L | 121 | -18 | -80 | -4 | 4.60 | .052 |
|  | Lingual (occipitotemporal) Gyrus | R | 120 | 18 | -82 | -2 | 4.13 | .053 |
| **High** |  |  |  |  |  |  |  |  |
|  | **N. S.** |  |  |  |  |  |  |  |
| **Interaction: Low > High** | |  |  |  |  |  |  |  |
|  | Anterior Cingulate Cortex | L | 216 | -8 | 22 | 26 | 5.83 | .013 |
|  | Vermis VI |  | 358 | 2 | -64 | -20 | 4.01 | .002 |

**Figure S4.** Contextual-informativeness (CI) effect after sentence-level matching on this parameter. The Low > High contrast reproduces the bilateral frontotemporal pattern reported in the main analysis, indicating that the CI effect is robust to matching on sentence-level informativeness, and is not driven by the original between-condition CI difference. For all analysis, the statistical threshold was set at p < .001 uncorrected, and only clusters larger than 87 voxels have been included (Monte-Carlo cluster-extent corrected at p < .05).

| **Table S8**: Peak MNI coordinates of clusters showing significant effects of contextual informativeness decrease in the sentence-level matched subsample (negative correlation). Cluster-level uncorrected p-value is provided for each cluster. | | | | | | | | |
| --- | --- | --- | --- | --- | --- | --- | --- | --- |
|  |  |  |  | Peak MNI coordinates | | |  |  |
|  | Anatomical Region (Peak) | Hem. | k | x | y | z | t | p (unc.) |
| **Low** |  |  |  |  |  |  |  |  |
|  | Supramarginal Gyrus | L | 445 | -52 | -52 | 28 | 5.66 | .001 |
|  | Middle Frontal Gyrus | R | 808 | 42 | 48 | 26 | 5.38 | <.001 |
|  | Inferior Frontal Gyrus | R | 421 | 32 | 24 | -8 | 5.03 | .002 |
|  | Medial Superior Frontal Gyrus | L | 989 | -4 | 32 | 42 | 4.84 | <.001 |
|  | Posterior Cingulate Cortex | L | 328 | -2 | -38 | 22 | 4.70 | .004 |
|  | Superior Frontal Gyrus | L | 299 | -22 | 52 | 0 | 4.67 | .006 |
|  | Lingual (occipitotemporal) Gyrus | L | 209 | -22 | -82 | -16 | 4.32 | .017 |
|  | Insula | L | 197 | -32 | 20 | -6 | 4.28 | .020 |
|  | Cerebellum Lobule VI | R | 146 | 26 | -78 | -18 | 4.21 | .041 |
|  | Precuneus | L | 169 | -12 | -66 | 32 | 3.79 | .030 |
| **High** |  |  |  |  |  |  |  |  |
|  | Middle Temporal Gyrus | R | 153 | 46 | -64 | -2 | 4.27 | .037 |
| **Interaction: Low > High** | |  |  |  |  |  |  |  |
|  | Insula | R | 728 | 32 | 22 | -8 | 5.70 | <.001 |
|  | Middle Frontal Gyrus | R | 970 | 42 | 48 | 24 | 5.69 | <.001 |
|  | Middle Cingulate Cortex | R | 520 | -2 | -24 | 28 | 5.22 | .001 |
|  | Supramarginal Gyrus | L | 312 | -52 | -52 | 28 | 5.04 | .005 |
|  | Insula | L | 250 | -32 | 20 | -6 | 4.91 | .010 |
|  | Superior Frontal Gyrus | L | 186 | -22 | 52 | 0 | 4.60 | .023 |
|  | Inferior Parietal Gyrus | R | 299 | 46 | -44 | 50 | 4.48 | .006 |
|  | Middle Cingulate Cortex | R | 1019 | 8 | 32 | 36 | 4.40 | <.001 |
|  | Precuneus | R | 761 | 12 | -62 | 32 | 4.25 | <.001 |
|  | Middle Frontal Gyrus | R | 160 | 40 | 22 | 36 | 4.08 | .034 |
|  | Cerebellum Crus I | L | 108 | -6 | -82 | -20 | 4.02 | .073 |
